# Maximum-parsimony haplotype frequencies inference based on a joint constrained sparse representation of pooled DNA

**DOI:** 10.1186/1471-2105-14-270

**Published:** 2013-09-08

**Authors:** Guido H Jajamovich, Alexandros Iliadis, Dimitris Anastassiou, Xiaodong Wang

**Affiliations:** 1 Translational and Molecular Imaging Institute, Icahn School of Medicine at Mount Sinai, New York NY 10029, USA; 2 Electrical Engineering Department, Columbia University, New York NY 10027, USA; 3 Center for Computational Biology and Bioinformatics, Columbia University, New York, NY 10027, USA

**Keywords:** DNA pools, Haplotype frequency estimation, Sparse representations, ADM

## Abstract

**Background:**

DNA pooling constitutes a cost effective alternative in genome wide association studies. In DNA pooling, equimolar amounts of DNA from different individuals are mixed into one sample and the frequency of each allele in each position is observed in a single genotype experiment. The identification of haplotype frequencies from pooled data in addition to single locus analysis is of separate interest within these studies as haplotypes could increase statistical power and provide additional insight.

**Results:**

We developed a method for maximum-parsimony haplotype frequency estimation from pooled DNA data based on the sparse representation of the DNA pools in a dictionary of haplotypes. Extensions to scenarios where data is noisy or even missing are also presented. The resulting method is first applied to simulated data based on the haplotypes and their associated frequencies of the AGT gene. We further evaluate our methodology on datasets consisting of SNPs from the first 7Mb of the HapMap CEU population. Noise and missing data were further introduced in the datasets in order to test the extensions of the proposed method. Both HIPPO and HAPLOPOOL were also applied to these datasets to compare performances.

**Conclusions:**

We evaluate our methodology on scenarios where pooling is more efficient relative to individual genotyping; that is, in datasets that contain pools with a small number of individuals. We show that in such scenarios our methodology outperforms state-of-the-art methods such as HIPPO and HAPLOPOOL.

## Background

In recent years large genome wide association studies have been considered a promising approach to identify disease genes. In these studies, which typically include thousands of individuals, a potential allele frequency difference for a specific marker between cases and controls could indicate an association between the marker and the disease.

Allele frequencies for cases and controls can be obtained either through individual genotyping or through DNA pooling. Although individual genotyping provides more accurate estimates of individual allele frequencies, as well as haplotypes which enable the study of genetic interactions, DNA pooling has been widely used as it can be more cost effective in genome wide association studies [1-6]. In genotype pooling, equimolar amounts of DNA from different individuals are mixed into one sample prior to the amplification and sequencing steps and the frequency of each allele in each position is given. Therefore, for pools of size *n*, the cost of genotyping is reduced by a factor of *n*[5].

As evident, one of the main concerns with the use of genotype pooling is genotype error. For a given pooled DNA sample, the standard deviation (SD) of the estimated allele frequency is between 1% and 4% [6]. However, as was argued by Kirkpatrick et al. [7] pooling errors have a greater effect on pools that contain a large number of individuals. To illustrate this point assume that *σ* is the SD of allele frequencies. After a genotype experiment, the ability of the clustering algorithms to correctly identify the number of each distinct allele depends on whether 2*σ* is smaller than the difference of allowable frequency calls. For example, in pools of two individuals where the difference between allowable frequency calls is 0.25 (0,0.25, 0.5, 0.75, 1), an accuracy of *σ*<0.125 will ensure a low rate of incorrect calls (<1*%*). For the same experiment, if pools of four individuals are considered then the difference of allowable frequencies is cut into half (0, 0.125, 0.25, 0.375, 0.5, 0.625, 0.75, 0.875, 1). Then, it is obvious that to get the same percentage of incorrect calls, *σ*, should be correspondingly halved. The situation quickly deteriorates for larger pool sizes.

Even though the main purpose of pooling is to screen alleles for potential discrepancies between cases and controls, estimating haplotype frequencies across a number of markers is also of interest with the pooled data as it can improve the power of detecting associations with disease. To facilitate haplotype-based association analysis it is necessary to estimate haplotype frequencies from pooled DNA data.

It has been claimed in the literature that pooling DNA samples is efficient for estimating haplotype frequencies. However, the results presented within the context of haplotype frequency estimation algorithms are largely numerical and they do not address the statistical properties and efficiency of the estimates being computed. In a recent study, Kuk et al. [8] addressed this issue and provided a general guideline on scenarios where pooling would be more efficient relative to individual genotyping. Instead of resorting to simulations, this study was based on theoretical analysis. For a fixed genotype cost, the authors have compared the maximum likelihood estimate based on pooled and individual genotype data. Their findings suggest that for the case of linkage equilibrium and non-rare allele, pooling begins to loose efficiency relative to no pooling when the number of loci is larger than 3 (2^3^ haplotypes with appreciable frequency). Factors such as Linkage Disequilibrium (LD) and rare alleles reduce the number of non-rare haplotypes appearing in the population and pooling could still remain more efficient either for a larger number of loci or when the pool size is kept considerably small, as suggested by Barratt et al. [9].

A variety of haplotype estimation methods from pooled genomic data have been proposed in the literature that fall into two large categories. The first category consists of methods that focus on a small number of markers but allow for considerably larger pool sizes while the second category of methods allows for a larger number of markers but for a small number of individuals per pool.

As haplotype frequency estimation from pooled genomic data can be seen as a missing data problem, it comes to no surprise that the majority of methods focusing on small pool sizes mainly contains methods that use the expectation-maximization (EM) algorithm for maximizing the multinomial likelihood [10-12]. Kirkpatrick et al. [7] suggested a perfect phylogeny method, HAPLOPOOL, that was supplemented with the EM algorithm and linear regression in order to combine haplotype segments and was shown to outperform competing EM algorithms.

Haplotype frequency estimation from large genotype pools was first addressed by Zhang et al. [13] using Poool and was further modified by Kuk et al. [14] resulting in the AEM algorithm. As the EM algorithm presents limitations in speed and difficulties with large pool sizes or long haplotypes, Kuk et al. [15] developed a fast collapsed method that trades performance but can handle larger datasets. Gasbarra et al.[16] introduced a haplotyping method for pooled DNA based on a continuous approximation of the multinomial distribution and a set of constraints (LinEq). The goal of the method is to perform haplotype inference incorporating prior database knowledge from databases such as HapMap. Finally, Pirinen introduced HIPPO [17], a Bayesian model for estimating the pooled haplotypes. HIPPO uses a multinormal approximation of the likelihood and a reversible-jump Markov chain Monte Carlo (RJMCMC) algorithm to estimate the existing haplotypes in the population and their frequencies. The HIPPO framework is also able to accommodate prior database knowledge for the existing haplotypes in the population and has demonstrated improvements in the performance over the AEM and LinEq methods.

There is also an equivalence between the haplotype frequencies estimation and the inference of relative abundances of species in mutagenomics studies. Kessner et al. [18] proposed an EM-based method based on individual sequence reads that can be used to deal with both scenarios. The haplotypes present in the pools are assumed to be known and need to be input to the method. Another EM method was proposed by Eskin et al. [19], where some individual genotypes are required in addition to the pooled sequence data. Amir et al. [20] proposed a method to reconstruct the abundance of each bacterium in a bacteria community by looking at a database of known 16S rRNA sequences and a single Sanger-sequence of the unknown mixture, by assuming that only a small set of bacteria are present within the set of bacteria with known 16S rRNA sequence.

In this study we present an algorithm for haplotype frequency estimation based on the maximum parsimony principle. A mathematical framework is presented where this principle is translated in a **joint** sparsity requirement and the frequency inference is performed using the alternating direction method (ADM) of multipliers. Our method focuses on datasets that have a small number of individuals per pool and a considerably large number of markers. We compare our method with the best performing methods from the two pooling algorithm categories as presented above, namely HIPPO and HAPLOPOOL. We have performed comparisons on a variety of marker and dataset sizes. All our comparisons represent scenarios for which, based on Kuk et al. [8], pooling is more efficient than individual genotyping. We show that our method demonstrates superior performance in terms of accuracy compared with state-of-the-art competing methods for almost all scenarios examined with special emphasis on scenarios where the number of loci is large.

## Results and discussions

In this section, first we describe the datasets and figures of merit used to evaluate the method. Then we present the results from comparing our method ADM to HIPPO and HAPLOPOOL.

All our comparisons were performed in scenarios were the use of pooling is potentially beneficial relative to no pooling according to the guidelines of Kuk et al. [8]. Our methodology specifically targets datasets that have a small number of individuals per pool and a large number of SNPs.

In real applications, it is very often the case that studies are performed in datasets for which partial knowledge of the existing haplotypes already exists (for example datasets from HAPMAP studied populations). This information could be used as a basis for an accurate definition of the haplotype dictionary matrix **H**, as will be defined in the Methods section, so that the number of possible haplotypes *M* is much smaller than the full set of allowable haplotypes. However, in order to evaluate the proposed method in the most general scenarios, no prior information is assumed and all possible haplotypes are considered.

The presented method is based on the augmented Lagrangian expansion of a constrained optimization problem which has an associated parameter *ρ*, as it will be shown in the Methods section. For all the results presented in this study, we have set $\rho =\frac{1}{ā}$ with ā being the average of the observed relative frequency of the allele 1 of the considered SNPs and pools. We have found experimentally that this choice of *ρ* achieves a good performance. Moreover, the ADM is an iterative method, which finalizes once a stopping criterion is met. For the results presented here, the *l*_2_-norm of the difference between the solution at step *k* and the solution at step *k*−1 over the *l*_2_-norm of the solution at step *k*−1 is compared to a tolerance parameter of 10^−20^. If the first term is smaller or *k*=8000, then the ADM stops and the solution at step *k* is presented.

### Datasets

To examine the performance of our methodology we have considered in our experiments real datasets for which estimates of the haplotype frequencies were already available and which cover a variety of dataset sizes.

We have first simulated data using the 10 loci haplotypes and their associated frequencies for the AGT gene considered in Yang et al. [12]. The haplotypes and their respective frequencies are given in Table 1. We have simulated datasets with different number of pools *O*=50, 75, 100 and 150. In each pool, each individual randomly selects a haplotype according to the distribution of haplotypes. For each pool size, we have created 100 datasets that were used as the datasets for our simulation.

**Table 1:** Haplotypes and frequencies for the AGT gene

**Haplotype**	**Frequency**
1 1 1 1 0 1 1 0 0 0	0.033
1 1 0 1 0 1 1 1 1 0	0.016
1 1 0 1 0 0 1 0 0 1	0.017
1 0 0 1 0 1 1 0 0 1	0.017
1 1 0 1 0 1 1 0 0 1	0.017
1 1 1 1 0 1 1 1 0 1	0.507
0 1 0 1 1 0 0 1 1 1	0.017
1 1 0 0 0 0 1 1 1 1	0.033
0 1 0 1 0 0 1 1 1 1	0.1
1 1 0 1 0 1 1 1 1 1	0.193
1 1 1 1 1 1 1 1 1 1	0.05

The second dataset consisted of SNPs from the first 7Mb (742 kb to 7124.8 kb) of the HapMap CEU population (HapMap 3 release 2- Phasing data (http://hapmap.ncbi.nlm.nih.gov/)). This chromosomal region was partitioned based on physical distance into disjoint blocks of 15 kb. The resulting blocks had a varying number of markers ranging from 2 to 28. For our purposes we have considered only the datasets that had more than 10 SNPs and less than 20 (which was the maximum number of loci so that HAPLOPOOL could produce estimates within a reasonable amount of time) which resulted in selecting a total of 80 blocks. On each block the parental haplotypes and their estimated frequencies were used as the true haplotype distribution. As in the previous cases in each block four different pool sizes were considered: *O*=50, 75, 100 and 150 pools.

### Performance criteria

Assume first that **g**= [*g*_1_⋯*g*_*M*_]^*T*^ is the gold standard haplotype frequency vector in a given dataset observed in the population and ***f***= [*f*_1_⋯*f*_*M*_]^*T*^ is the predicted haplotype frequency vector from a given method. To compare the performance of different methodologies we have considered two criteria:

*χ*^2^ distance: The *χ*^2^ distance between the two distributions **g** and ***f*** is defined as $\chi^{2}(\;\;f,g)\;=\;\overset{M}{\underset{i=1,g_{i}\neq 0}{\sum }}{\left(\;\;f_{i}-g_{i}\right)}^{2}/g_{i}$ where only the terms with non-zero haplotype frequency vector *g*_*i*_ are considered.

*l*_1_ distance: The *l*_1_ distance between the two distributions is defined as $l_{1}(\;\;f,g)=\overset{M}{\underset{i=1}{\sum }}|\;\;f_{i}-g_{i}|$.

### Frequency estimation

**Figure 1:**
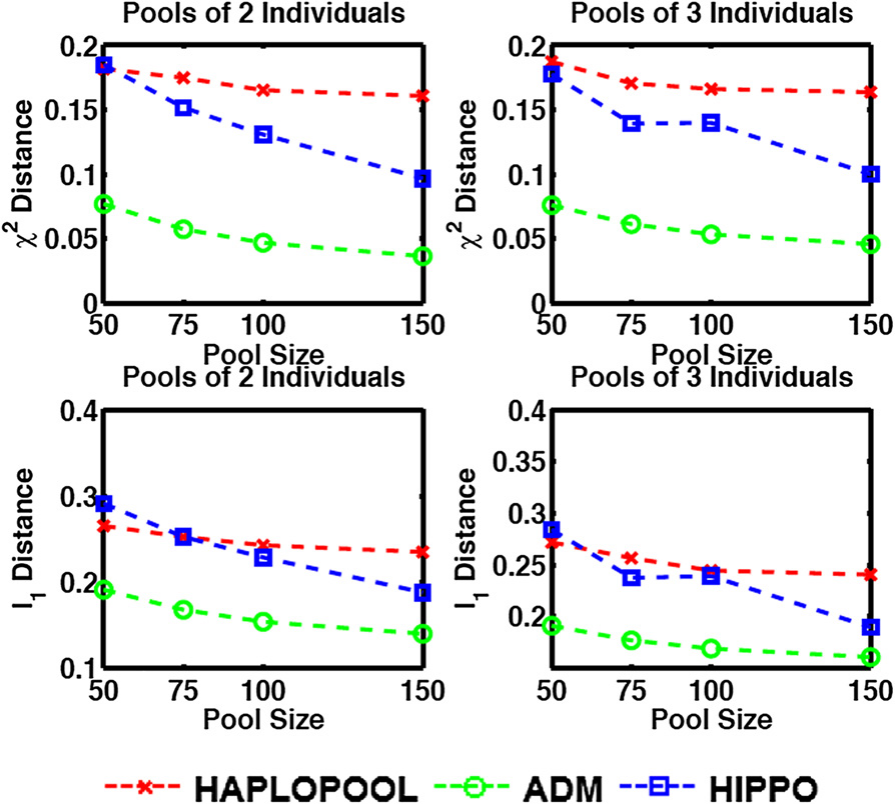
Measures of performance of HAPLOPOOL, HIPPO and ADM applied to the AGT gene dataset.

**Figure 2:**
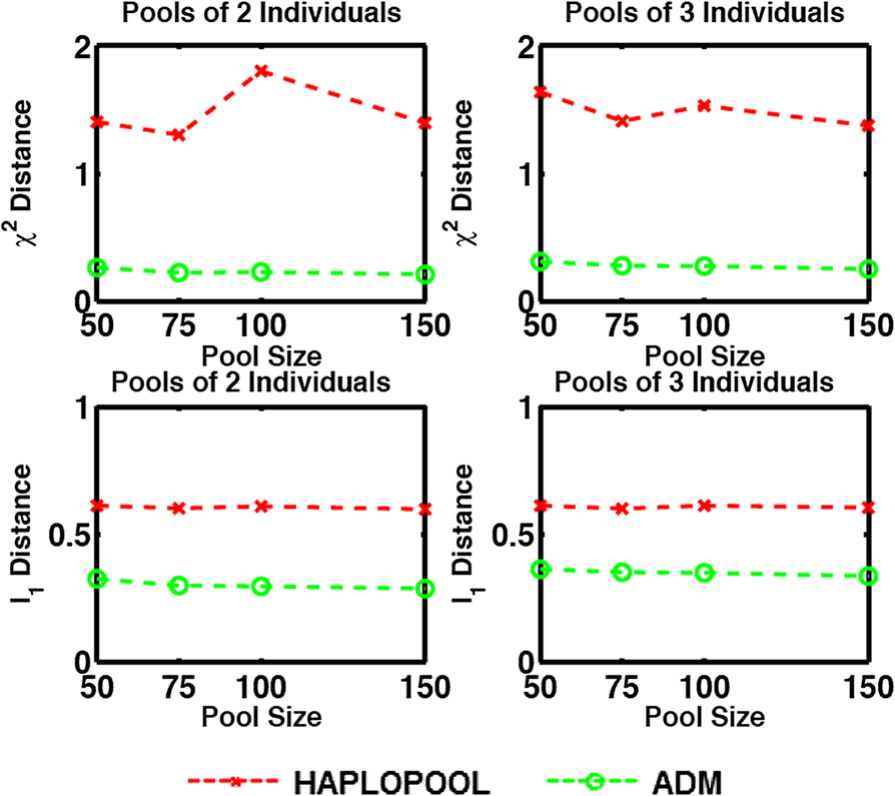
Measures of performance of HAPLOPOOL and ADM applied to the HapMap dataset.

We have examined the accuracy of our method and compared it against HIPPO and HAPLOPOOL on the AGT gene and HapMap datasets described in our previous subsection. The performance of the methods is shown in Figures 1 and 2. For the 10 loci dataset the results shown are the average *χ*^2^ and *l*_1_ distance from a 100 simulation experiments. We can see that ADM demonstrated superior performance for both figures of merit (Figure 1).

For the HapMap dataset (Figure 2) only ADM and HAPLOPOOL were evaluated since the maximum number of loci HIPPO can handle is 10. At the same time, even though HAPLOPOOL can in principle handle larger datasets, we restricted our comparisons to datasets between 10 and 20 loci due to excessive computational time.

From our experiments we can also see that the number of pools also affected accuracy. All algorithms demonstrated improved performance with increasing number of pools in the dataset.

### Noise and missing data

We have further evaluated the performance of our method in the presence of measurement error. We have simulated genotyping error by adding a Gaussian error with SD *σ* to each called allele frequency. In particular, if we denote the correct allele frequency at SNP *j* in pool *i* as *c*_*ij*_, then the perturbed allele frequency is given by ${ĉ}_{\text{ij}}=c_{\text{ij}}+x$ where *x*∼*N*(0,*σ*^2^). To obtain the allele counts we discretize each allele frequency to the closest allowed frequency depending on the number of individuals per pool and obtain the allele counts accordingly.

**Figure 3:**
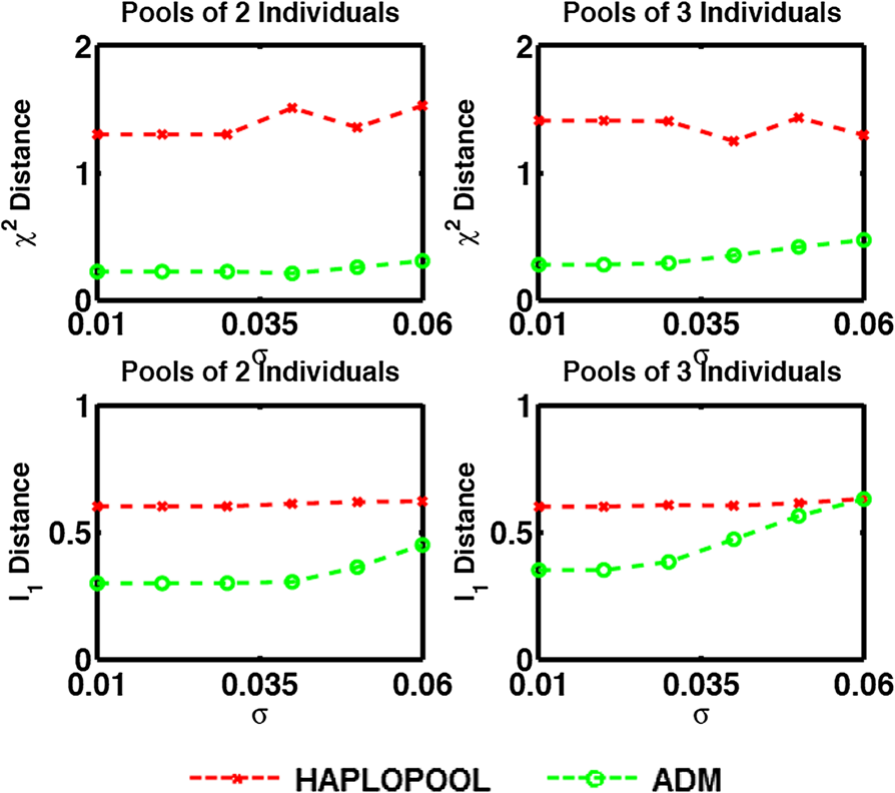
Measures of performance of HAPLOPOOL and ADM applied to the HapMap dataset with noisy observations.

We have selected the values for *σ* so that they represent realistic scenarios and thus ranging between 0 (no measurement error) and 0.06 [5-7]. The ADM method has a parameter *δ* that takes into account the presence of noise which could be set to be a function of *σ*. However, the parameter was set to *δ*=0.1 for all tested *σ* as the variance of noise in the sample is not assumed to be known in advance. The results are shown in Figure 3. We give the results only when the number of pools is 75 but the shape of the figures is similar for the remaining pool sizes examined in our previous examples.

We can see that ADM demonstrates superior performance compared to competing methods and, as expected, its performance deteriorates with increasing noise levels. The results also demonstrate the fact that pooling errors affect more pools that contain a large number of individuals. The reason is, as has been noted before, that in smaller pools the gap between allowable frequency calls is much larger resulting in a smaller percentage of miscalled allele counts and thus in better frequency estimates.

**Figure 4:**
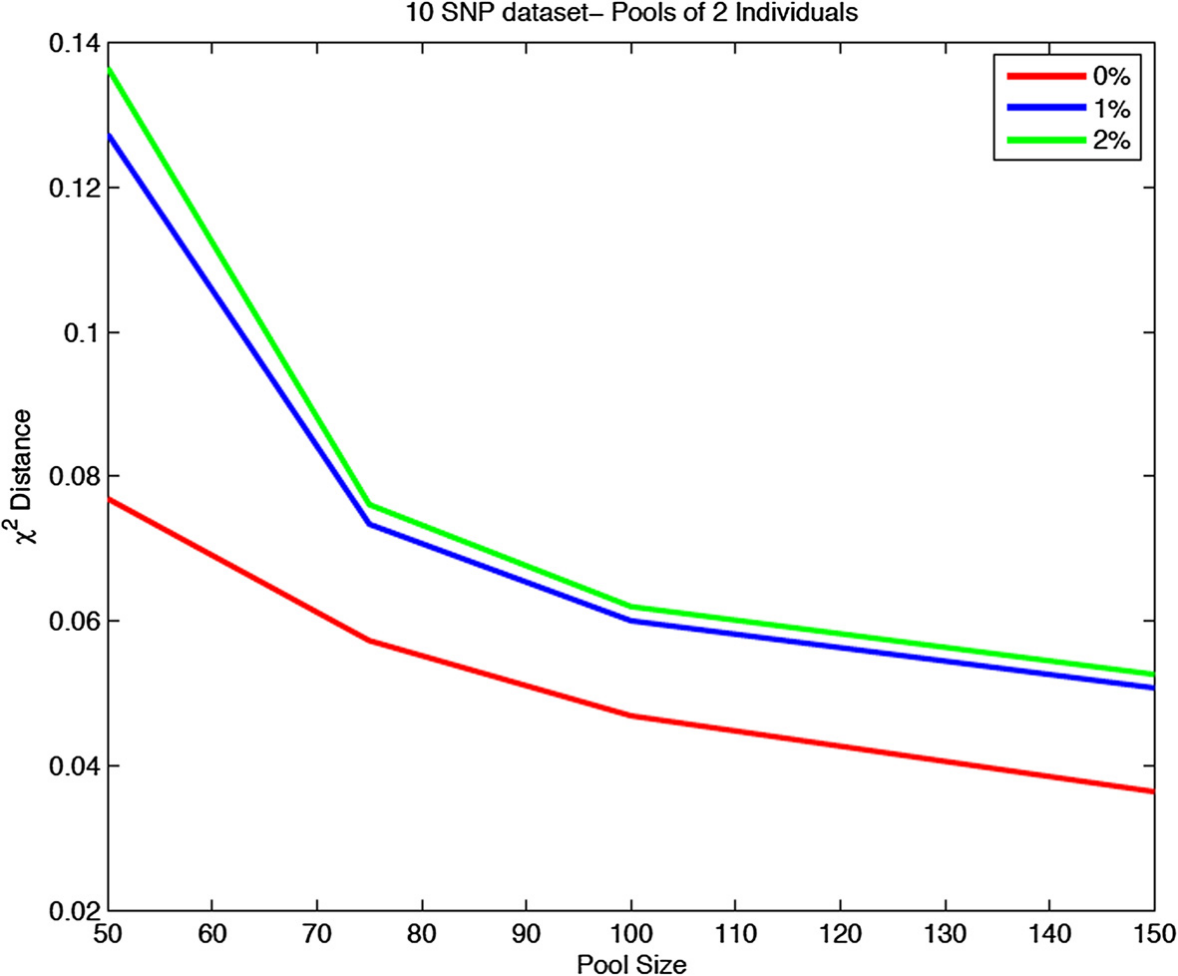
Measures of performance of ADM applied to the HapMap dataset with missing observations.

We have further set a realistic percentage of SNPs to be missing (1% and 2% per dataset) and demonstrated the accuracy of our modified methodology. As shown in Figure 4, the performance of our method slightly deteriorates with an increase in the proportion of missing SNPs while, similar to the previous scenarios examined, the accuracy increases with increasing pool size.

## Conclusions

In this study we have presented a method for estimating haplotype frequencies from pooled data based on the maximum parsimony principle. A novel mathematical framework is introduced where this principle is translated to finding a sparse representation of the observed DNA pools in a dictionary of haplotypes. This leads to an optimization problem that is solved with the alternating direction method of multipliers. The proposed method is also extended to scenarios where noisy and missing data is present in the considered DNA pools, and is able to process pools with a large number of SNPs.

Numerical experiments using synthetic and real data have shown improved performance with respect to the best of the haplotype frequency inference methods. In particular, the proposed ADM method is an efficient method that performs better than other methods such as HIPPO and HAPLOPOOL in the considered datasets consisting of pools with a small number of individuals and a large number of markers.

## Methods

### Overview

This section provides a description of the proposed method for haplotype frequencies inference based on the maximum-parsimony principle. The method seeks to discover the frequencies of the haplotypes present in a population given the observed relative frequencies of each allele in each DNA pool. In order to obtain a biological meaningful estimation, the proposed method makes use of the maximum-parsimony principle which attempts to minimize the total number of haplotypes observed in the sample [21].

Each pool has an associated vector of observed relative frequencies that, with the proposed mathematical framework, can be expressed as the linear combination of haplotypes of a dictionary. This dictionary of haplotypes can be constructed using information from external databases [16] or, in the most general case where such information is not available, all possible haplotypes need to be considered. Each vector of observed relative frequencies should be reconstructed with the minimum number of distint haplotypes in the dictionary according to the maximum-parsimony principle. Moreover, as there are more than one pool available, the set of used haplotypes needs to be selected to explain all pools **jointly**.

This framework for haplotype frequencies estimation leads to a joint constrained sparse optimization problem. This kind of optimization problem has been studied in the compressed sensing literature, where the alternating direction method (ADM) of multipliers has been proposed to find the corresponding solution. The proposed method makes use of the ADM adapted to the haplotype frequencies estimation.

### Maximum-parsimony haplotype frequencies inference framework

The proposed method estimates the frequencies of haplotypes consisting of *L* diallelic loci residing on a narrow chromosomal interval. In each locus, only two out of the four different nucleotides can be found in a large percentage of the population. The most common nucleotide in that locus is called the wild-type and is encoded with a 0 and the other nucleotide is the mutant and is encoded with a 1. We define the haplotype dictionary matrix **H** as an *L*×*M* matrix containing the *M* possible distinct haplotypes as its columns. To obtain **H**, we can use information from external databases [16] or, when this information is not available, all possible haplotypes of length *L* must be considered. We consider *O* pools, where each pool consists of *n*_*i*_ individuals (*i*=1,⋯,*O*) and therefore, there are 2*n*_*i*_ haplotypes in each pool. Moreover, we define $p^{i}≜{\left[p_{1}^{i}\;\cdots p_{M}^{i}\right]}^{T}$, where $p_{j}^{i}$ is the unknown proportion of the *j* haplotype in the *i*-th pool, and $a^{i}≜{\left[a_{1}^{i}\;\cdots a_{L}^{i}\right]}^{T}$, with $a_{l}^{i}$ is the observed relative frequency of the allele 1 in the *i*-th pool for the *l*-th SNP. Then, the unknown vectors **p**^*i*^ satisfy

(1) $$a^{i}=Hp^{i},\;p^{i}≽0,\;∥p^{i}{∥}_{1}=1,$$

where $∥p^{i}{∥}_{1}≜\overset{M}{\underset{j=1}{\sum }}|p_{j}^{i}|$ is the *l*_1_-norm of the vector **p**^*i*^. Since **p**^*i*^≽***0***, we have $∥p^{i}{∥}_{1}=\overset{M}{\underset{j=1}{\sum }}p_{j}^{i}$; that is, the *l*_1_-norm is the sum of the proportions which needs to be 1. Each proportion $p_{j}^{i}$ can only be discrete multiples of the basic unit of $\frac{1}{2n_{i}}$; that is, $p_{j}^{i}$ takes values in the set $\left\{0,\frac{1}{2n_{i}},\cdots \;,1\right\}$, but as measurements contain noise, we relax this condition and allow each proportion to take any value in the interval [0,1] [16].

Then the haplotype frequency estimation problem can be stated as follows: Given the observed relative frequencies of the alleles **a**^*i*^, *i*=1,⋯,*O*, infer the proportions of the haplotypes **p**^*i*^, *i*=1,⋯,*O*, in every pool. The dimension of each relative frequency of the alleles **a**^*i*^ is *L*, while the dimension of the unknown proportion vector **p**^*i*^ is *M*, where generally *M*≫*L*; that is, the estimation task is an ill-posed inverse problem and side information is needed to complete this task. In particular, in this paper, we make use of the *maximum parsimony principle*. This principle states that the number of different haplotypes that explains all the observed relative frequency vectors **a**^*i*^ should be as small as possible. Therefore, the maximum parsimony haplotype inference problem is stated as follows. Given the set {**a**_*i*_,*i*=1,…,*O*} of observed relative frequency vectors of *i* pools with *n*_*i*_ subjects and for *L* loci, we aim at inferring the vector of proportions {**p**_*i*_,*i*=1,…,*O*} that is composed of the minimum number of distinct haplotypes. From the point of view of Eq. (1), the maximum parsimony principle can be translated as using as few columns of **H** as possible to explain all the observed frequency vectors **a**^*i*^.

### Haplotype frequencies inference based on a joint constrained sparse representation of pooled DNA

We define $X≜\;[p^{1}\cdots p^{O}]$ as the unknown matrix containing the proportions of the haplotypes for the *O* pools, and equivalently, $A≜\;[a^{1}\cdots a^{O}]$. Then, taking into account all pools, (1) becomes

(2) $$A=HX,\;X≽0,\;1^{T}X=1^{T},$$

where $1≜\;{\left[\;1\;\cdots 1\right]}^{T}$. The maximum parsimony principle dictates that the inferred proportions $\hat{X}$ that satisfies (2) utilizes the least number of columns of matrix **H**. This is equivalent to requiring the inferred solution $\hat{X}$ to have row-sparsity; that is, let **x**^*i*^ and **x**_*j*_ be the *i*-th row and the *j*-th column of matrix **X**, respectively and define a vector **e**(**X**) containing the energy of each row of matrix **X**, i.e., $e(X)≜{\left[e(x^{1})\;e(x^{2})\cdots e(x^{M})\right]}^{T}$ with *e*(**x**^*i*^)=∥**x**^*i*^∥_2_, then row-sparsity implies finding the solution to the following optimization problem

(3) $$\begin{array}{l} \underset{X}{\min}\;{∥e(X)∥}_{0}\\ \;\mathrm{s.t.}\;\left\{\begin{array}{l} \;HX=A\\ \;1^{T}X=1^{T}\\ \;X≽0, \end{array}\right) \end{array}$$

where ∥**e**(**X**)∥_0_ is the *l*_0_ norm of the vector **e**(**X**) and corresponds to the number of non-zero components of the vector. This means that the solution will have as many all-zero rows as possible.

However, minimizing an *l*_0_ norm is computational intractable as it involves solving a combinatorial problem. One option well studied in the compressed sensing literature is to replace the *l*_0_ norm with the *l*_1_ norm, as it promotes sparsity and leads to more tractable solutions. Then, the inference, in our case, becomes the solution to

(4) $$\begin{array}{l} \underset{X}{\min}\;{∥e(X)∥}_{1}\\ \;\mathrm{s.t.}\;\left\{\begin{array}{l} \;HX=A\\ \;1^{T}X=1^{T}\\ \;X≽0. \end{array}\right) \end{array}$$

This matrix problem lies within the convex optimization framework. In the most general case where there is no prior information regarding the possible haplotypes to be considered, the size of the matrix **H** grows exponentially with the number of SNPs. In what follows we present an efficient method to find the solution to (4) by means of the alternating direction method (ADM) of multipliers. The ADM proceeds to solve local small problems in order to uncover the global solution to the problem with proven convergence; that is, the ADM is guaranteed to find the optimal solution to (4) [22]. We first briefly describe the ADM in its general form and then we show how (4) can be solved with the ADM.

#### Alternating direction method of multipliers

Given two convex functions $f:{\mathbb{R}}^{m_{1}}\to \mathbb{R}$ and $g:{\mathbb{R}}^{m_{2}}\to \mathbb{R}$, the alternating direction method of multiplier is used in order to find the solution to the following optimization problem of two sets of variables $x\in {\mathbb{R}}^{m_{1}}$ and $z\in {\mathbb{R}}^{m_{2}}$

(5) $$\begin{array}{cc} \underset{x,z}{\min} & \;f(x)+g(z)\\ \mathrm{s.t.} & \;Cx+Dz=e, \end{array}$$

with $C\in {\mathbb{R}}^{p\times m_{1}}$, $D\in {\mathbb{R}}^{p\times m_{2}}$, and $e\in {\mathbb{R}}^{p}$.

For *ρ*>0, the augmented Lagrangian of (5) is given by [22]

(6) $$\begin{array}{l} \;L_{\rho }(x,z,y)=f(x)+g(z)+y^{T}\left(Cx+Dz-e\right)\\ \;+\rho {∥Cx+Dz-e∥}_{2}^{2} \end{array}$$

Minimizing (6) with respect to **x** and **z** jointly is usually not tractable. Instead, the alternating direction method of multiplier proceeds to iterate minimizing (6) over **x** for a fixed **z**, followed by the minimization of (6) with respect to **z** for a fixed **x** and a dual variable update; that is, let $u≜\frac{1}{\rho }y$, Table 2 illustrates the steps involved. It is seen in this table that the global solution to (5) is found by solving the local small problems of steps 6 and 7.

**Table 2:** Alternating direction method of multipliers

1	Set *k *= 0
2	Set *ρ *> 0
3	Initialize ***x***^0^, ***z***^0^ and ***u***^0^
4	Repeat
5	*k *= *k *+ 1
6	$x^{k+1}≜\arg\underset{x}{\min}\left(f(x)+\frac{\rho }{2}\overset{2}{\underset{2}{∥Cx+Dz^{k}-e+u^{k})∥}}\right)$
7	$z^{k+1}≜\arg\underset{z}{\min}\left(g(z)+\frac{\rho }{2}\overset{2}{\underset{2}{∥Cx^{k+1}+Dz-e+u^{k})∥}}\right)$
8	$u^{k+1}≜u^{k}+\left(Cx^{k+1}+Dz^{k+1}-e\right)$
9	until convergence

### Joint constrained sparse haplotype frequency estimation algorithm

Introducing the *M*×*O* matrix **Z**, (4) can be restated in order to apply the ADM and obtain closed-form expressions for the local minimization steps as follows

(7) $$\begin{array}{l} \underset{X,Z}{\min}\;{∥e(Z)∥}_{1}\\ \;\mathrm{s.t.}\;\left\{\begin{array}{l} \;HX=A\\ \;1^{T}X=1^{T}\\ \;Z≽0\\ \;X=Z. \end{array}\right) \end{array}$$

This optimization problem can be restated in the framework of (5) by defining

$$\begin{array}{cc} x & ≜\left(\begin{array}{l} x_{1}\\ \vdots \\ x_{O} \end{array}\right),\;\;z≜\left(\begin{array}{l} z_{1}\\ \vdots \\ z_{O} \end{array}\right),\;\;e≜\left(\begin{array}{l} a_{1}\\ \vdots \\ a_{O}\\ 0_{O\cdot M\times 1} \end{array}\right),\\ C & ≜\left(\begin{array}{l} I_{O}⊗H\\ I_{O\cdot M} \end{array}\right),\;\;D≜\left(\begin{array}{l} 0_{O\cdot L\times O\cdot M}\\ -I_{O\cdot M} \end{array}\right), \end{array}$$

where ⊗ is the Kronecker product, ***0***_*O*·*M*×1_ is an *O*·*M*×1 zero vector, **I**_*O*_ is the *O*×*O* identity matrix, **I**_*O*·*M*_ is the *O*·*M*×*O*·*M* identity matrix and ***0***_*O*·*L*×*O*·*M*_ is an *O*·*L*×*O*·*M* zero matrix, and

(8) $$\begin{array}{l} f(x)≜U_{\left(Ex-1\right)}(x)\\ g(z)≜\overset{M}{\underset{i=1}{\sum }}∥z_{g_{i}}{∥}_{2}+U_{\left(z\geq 0\right)}(z), \end{array}$$

where $E≜\left(\begin{array}{l} 1^{T}\\ \ddots \\ 1^{T} \end{array}\right)$, *U*_*S*_ is the indicator function of the set *S* (that is, *U*_*S*_(**x**)=0 if **x**∈*S* and *∞* otherwise), and $z_{g_{i}}$ is the vector of components in **z** that correspond to the *i*-th row of matrix **Z**.

With these definitions, the steps of the ADM in Table 2 lead to the joint constrained sparse haplotype frequency estimation algorithm of Table 3. The Shrink function is an operation applied row-wise to the matrix input and is given by

(9) $$\text{Shrink}(r,a)≜\max\left(∥r{∥}_{2}-a,0\right)\frac{r}{∥r{∥}_{2}},$$

**Table 3:** Joint constrained sparse haplotype frequency estimation algorithm

1	Set *k *= 0
2	Set *ρ *> 0
3	Set **X**^0^ = ***0***, **Z**^0^ = ***0***, $U_{1}^{0}=0$, $U_{2}^{0}=0$
4	**U**_4_ = (**I**−**H**^*T*^(**I**+**HH**^*T*^)^−1^**H**)
5	Repeat
6	*k *= *k *+ 1
7	$u_{3}^{T}=\frac{1^{T}U_{4}\left(H^{H}\left(U_{2}^{k}-A\right)+U_{1}^{k}-Z^{k}\right)-1^{T}}{1^{T}U_{4}1}$
8	$X^{k+1}=U_{4}\left(U_{1}^{k}-Z^{k}+H^{T}(U_{2}^{k}-A)-1u_{3}^{T}\right)$
9	$Z^{k+1}=\max\left(\text{Shrink}\left(X^{k+1}+\frac{1}{\rho }U_{1}^{k},\frac{1}{\rho }\right),0\right)$
10	$U_{1}^{k+1}=U_{1}^{k}+X^{k+1}-Z^{k+1}$
11	$U_{2}^{k+1}=U_{2}^{k}+HX^{k+1}-A$
12	until convergence

the max operation of step 9 is component-wise, and $0≜{\left[\;0\;\cdots 0\right]}^{T}$.

### Extensions

#### Noisy data

Measurement errors in determining allele frequencies are considerable in DNA pools, presenting a variance between 0.02 and 0.04 [5,7]. This means that imposing the constraint **HX**=**A** is too restrictive and can be relaxed in order to take the measurement noise into account. In particular, we introduce a parameter *δ*, and we propose to find the maximum-parsimony solution by solving the following optimization problem.

(10) $$\begin{array}{l} \underset{X}{\min}\;{∥e(X)∥}_{1}\\ \;\mathrm{s.t.}\;\left\{\begin{array}{c} \sqrt{\overset{O}{\underset{i=1}{\sum }}∥a^{i}-Hp^{i}{∥}_{2}^{2}}\leq \delta \\ 1^{T}X=1^{T}\\ X≽0. \end{array}\right) \end{array}$$

Introducing the *M*×*O* matrix **Z**_1_ and the *L*×*O* matrix **Z**_2_, the ADM method can be used to solve (10), by solving the equivalent problem

(11) $$\begin{array}{l} \underset{Z_{1},Z_{2},X}{\min}\;{∥e(Z_{1})∥}_{1}\\ \;\mathrm{s.t.}\;\left\{\begin{array}{l} \;\sqrt{\overset{O}{\underset{i=1}{\sum }}∥a^{i}-z_{2}^{i}{∥}_{2}^{2}}\leq \delta \\ \;1^{T}X=1^{T}\\ \;Z_{1}≽0\\ \;Z_{1}=X\\ \;Z_{2}=HX, \end{array}\right) \end{array}$$

where $z_{2}^{i}$ is the *i*-th column of matrix **Z**_2_. This simple transformation allows us to obtain closed-form expressions for the local minimization steps of the ADM.

The maximum parsimony solution to the haplotype frequency inference estimation with noisy observations can be found by following the steps illustrated in Table 4, where $x_{i}^{k+1}$ and $u_{2,i}^{k}$ correspond to the *i*-th column of **X**^*k*+1^ and $U_{2}^{k}$ respectively, and

(12) $$\;\begin{array}{l} Z_{2}^{k+1}=\text{proj}(H,X^{k+1},U_{2}^{k},A,\delta )\\ \;=\left\{\begin{array}{l} \;HX^{k+1}+U_{2}^{k}\;\text{if}\sqrt{\overset{O}{\underset{i=1}{\sum }}\;∥Hx_{i}^{k+1}\;+u_{2,i}-a_{i}{∥}_{2}}\leq \;\delta \\ \;A+\frac{HX^{k+1}+U_{2}^{k}-A}{\sqrt{\overset{O}{\underset{i=1}{\sum }}∥Hx_{i}^{k+1}+u_{2,i}-a_{i}{∥}_{2}}}\delta \;\text{otherwise} \end{array}\right) \end{array}$$

**Table 4:** Joint constrained sparse haplotype frequency estimation algorithm in the presence of noisy measurements

1	Set *k * = 0
2	Set *ρ * > 0
3	Set **X**^0^ = ***0***, **Z**^0^ = ***0***, $U_{1}^{0}=0$, $U_{2}^{0}=0$
4	**U**_4_ = (**I**−**H**^*T*^(**I**+**HH**^*T*^)^−1^**H**)
5	Repeat
6	*k *= *k *+ 1
7	$u_{3}^{T}=\frac{1^{T}U_{4}\left(H^{H}\left(U_{2}^{k}-Z_{1}^{k}\right)+U_{1}-Z_{2}^{k}\right)-1^{T}}{1^{T}U_{4}1}$
8	$X^{k+1}=U_{4}\left(U_{1}^{k}-Z_{1}^{k}+H^{T}(U_{2}^{k}-Z_{2}^{k})-1u_{3}^{T}\right)$
9	$Z_{1}^{k+1}=\max\left(\text{Shrink}\left(X^{k+1}+\frac{1}{\rho }U_{1}^{k},\frac{1}{\rho }\right),0\right)$
10	$Z_{2}^{k+1}=\text{proj}(H,X^{k+1},U_{2}^{k},A,\delta )$
11	$U_{1}^{k+1}=U_{1}^{k}+X^{k+1}-Z_{1}^{k+1}$
12	$U_{2}^{k+1}=U_{2}^{k}+HX^{k+1}-Z_{2}^{k+1}$
13	until convergence

#### Missing data

Errors often occur during the genotyping process, and the data at some loci might not have been observed. We present modifications to the algorithms to perform haplotype inference in the presence of missing data. We assume that it is known a priori where the genotype information is missing for each genotype of each individual.

The presence of missing data in a genotype of a given pool imply a smaller number of constraints. Let ${\tilde{a}}_{i}$ be the observed relative frequency vector where all the loci with missing information have been removed, and **H**_*i*_ the matrix with all the rows corresponding to those loci removed. Notice that different pools present missing information in different loci, making the matrix dependant on the considered individual.

The solution to the haplotype inference problem can be found by solving

(13) $$\begin{array}{l} \underset{X}{\min}\;{∥e(X)∥}_{1}\\ \;\mathrm{s.t.}\;\left\{\begin{array}{l} \;H_{i}x_{i}={\tilde{a}}_{i}\;i=1,\cdots \;,O\\ \;1^{T}X=1^{T}\\ \;X≽0. \end{array}\right) \end{array}$$

The ADM is also used to find the solution to this optimization problem, and the resulting steps to find the haplotype frequency estimation are shown in Table 5.

**Table 5:** Joint constrained sparse haplotype frequency estimation algorithm with missing data

1	Set *k *= 0
2	Set *ρ *> 0
3	Set **X**^0^ = ***0***, **Z**^0^ = ***0***, $U_{1}^{0}=0$, $U_{2}^{0}=0$
4:	**U**_4_ = (**I**−**H**^*T*^(**I**+**HH**^*T*^)^−1^**H**)
5	Repeat
6	*k *= *k *+ 1
7	For *i * = 1,⋯,*O*
8	$u_{3,i}=\frac{1^{T}U_{4}\left(H_{i}^{T}\left(u_{2,i}^{k}-{\tilde{a}}_{i}\right)+u_{i}^{1}-z_{i}^{k}\right)-1}{1^{T}U_{4}1}$
9	$x_{i}^{k+1}=U_{4}\left(u_{1,i}^{k}-z_{i}^{k}+H_{i}^{T}(u_{2,i}^{k}-\tilde{a_{i}})-u_{3,i}1\right)$
10	end for;
11	$Z^{k+1}=\max\left(\text{Shrink}\left(X^{k+1}+\frac{1}{\rho }U_{1}^{k},\frac{1}{\rho }\right),0\right)$
12	$U_{1}^{k+1}=U_{1}^{k}+X^{k+1}-Z^{k+1}$
13	For *i * = 1,⋯,*O*
14	$u_{2,i}^{k+1}=u_{2,i}^{k}+H_{i}x_{i}^{k+1}-{\tilde{a}}_{i}$
15	end for
16	until convergence

#### Large number of SNPs

When the number of SNPs is large, the size of the matrix **H** increases dramatically. One approach for this case is to partition the data into blocks and process one block at a time. After all blocks are processed, a ligation process is performed to obtain the final result. We adopt the partition-ligation (PL) method [23] for haplotype frequency estimation.

The PL method starts with the partition phase. The vectors of observed relative frequencies **a**_*i*_,*i*=1,⋯,*O* is divided into *Q* non-overlapping and non-empty sets that cover all of the vectors. Each set contains segments from the same SNP loci for all individuals. Let $\left\{G_{q_{1}^{1}:q_{2}^{1}},G_{q_{1}^{2}:q_{2}^{2}}\cdots \;,G_{q_{1}^{Q}:q_{2}^{Q}}\right\}$ be the partitioned sets of relative frequency vectors, where the *i*-th subset $G_{q_{1}^{i}:q_{2}^{i}}$ contains the relative frequencies for SNP locus $q_{1}^{i}$ to $q_{2}^{i}$ for all *N* individuals. We impose that the first locus of the first set be the first locus of the complete genotype, i.e., $q_{1}^{1}=1$. Moreover, each set is adjacent to the previous one, i.e., $q_{1}^{i}=q_{2}^{i-1}+1$ for *i*={2⋯*Q*}. Notice that as we need to cover all loci, the last locus for the last set is $q_{2}^{Q}=L$. For each set $G_{q_{1}^{i}:\;q_{2}^{i}}$, the haplotypes frequencies are inferred using our algorithm, which outputs a small set of haplotypes frequencies.

Then, the PL proceeds to a ligation phase, where adjacent sets are merged to obtain a new partition of the data, with $\left\lceil \frac{Q}{2}\right\rceil$ sets, e.g., when merging the (2*i*)-th set with the (2*i*+1)-th set, the resulting set consists of the observed frequencies for all individuals between locus $q_{1}^{2i}$ and $q_{2}^{2i+1}$. For each merged set $G_{q_{1}^{2i}:\;q_{2}^{2i+1}}$, we run the haplotype inference algorithm again, but restricting **H** to contain every possible concatenations of the haplotypes of the (2*i*)-th set with the haplotypes of the (2*i*+1)-th set that have non-zero estimated frequencies. The process continues until there is only one set of relative frequencies and the haplotype frequencies inference algorithm is finally applied to this set.

In order to use the PL method, we need to determine an initial partition of the data. Therefore, we need to specify the number of partitions *Q* and the length of each partition or equivalently, the initial locus of each partition, i.e., ${\left\{q_{1}^{i}\right\}}_{i=1\cdots Q}$. A simple and low-cost way of setting the initial loci ${\left\{q_{1}^{i}\right\}}_{i=1\cdots Q}$ is to fix each block to be of equal length. Then, given an upper bound *W* on the length for each initial block, the number of blocks is $Q=\lceil \frac{L}{W}\rceil$.

## Availability of supporting data

Our method is available for download at http://www.ee.columbia.edu/~guido/ADM/.

## Competing interests

The authors declare that they have no competing interests.

## Authors’ contributions

XW and DA conceived of the study. GHJ, AI, DA and XW participated in the design of the study. GHJ and AI performed the computer experiments and wrote the first draft of the manuscript. All authors read and approved the final manuscript.
